# Detection of unrecorded environmental challenges in high-frequency recorded traits, and genetic determinism of resilience to challenge, with an application on feed intake in lambs

**DOI:** 10.1186/s12711-020-00595-x

**Published:** 2021-01-06

**Authors:** Carolina Andrea Garcia-Baccino, Christel Marie-Etancelin, Flavie Tortereau, Didier Marcon, Jean-Louis Weisbecker, Andrés Legarra

**Affiliations:** 1 GenPhySE, Université de Toulouse, INRAE, ENVT, 31326 Castanet-Tolosan, France; 2 Facultad de Agronomía, Universidad de Buenos Aires, 1417 Buenos Aires, Argentina; 3 Unité Expérimentale INRAE, Domaine de La Sapinière, INRAE, 18390 Osmoy, France

## Abstract

**Background:**

Resilient animals can remain productive under different environmental conditions. Rearing in increasingly heterogeneous environmental conditions increases the need of selecting resilient animals. Detection of environmental challenges that affect an entire population can provide a unique opportunity to select animals that are more resilient to these events. The objective of this study was two-fold: (1) to present a simple and practical data-driven approach to estimate the probability that, at a given date, an unrecorded environmental challenge occurred; and (2) to evaluate the genetic determinism of resilience to such events.

**Methods:**

Our method consists of inferring the existence of highly variable days (indicator of environmental challenges) via mixture models applied to frequently recorded phenotypic measures and then using the inferred probabilities of the occurrence of an environmental challenge in a reaction norm model to evaluate the genetic determinism of resilience to these events. These probabilities are estimated for each day (or other time frame). We illustrate the method by using an ovine dataset with daily feed intake (DFI) records.

**Results:**

Using the proposed method, we estimated the probability of the occurrence of an unrecorded environmental challenge, which proved to be informative and useful for inclusion as a covariate in a reaction norm animal model. We estimated the breeding values for sensitivity of the genetic potential for DFI of animals to environmental challenges. The level and slope of the reaction norm were negatively correlated (− 0.46 ± 0.21).

**Conclusions:**

Our method is promising and appears to be viable to identify unrecorded events of environmental challenges, which is useful when selecting resilient animals and only productive data are available. It can be generalized to a wide variety of phenotypic records from different species and used with large datasets. The negative correlation between level and slope indicates that a hypothetical selection for increased DFI may not be optimal depending on the presence or absence of stress. We observed a reranking of individuals along the environmental gradient and low genetic correlations between extreme environmental conditions. These results confirm the existence of a G $\times$ E interaction and show that the best animals in one environmental condition are not the best in another one.

## Background

Resilience is the capacity of an animal to be minimally affected by disturbances or to rapidly return to its state prior exposure to a disturbance or environmental challenge [1, 2]. This concept is sometimes confused with robustness but resilience and robustness are different. Robustness, in the context of intensive livestock production, refers to the combination of a high production potential with high resilience to external stressors, which allows for unproblematic expression of that production potential in a wide variety of environmental conditions. Thus, robustness is very similar to general resilience to a variety of stressors, which focus in particular on high-performance genotypes [3, 4]. Further differences between these two concepts are described in [1]. Robustness is a difficult phenotype to properly characterize because it is a complex trait that is composed of multiple components, including dynamic elements such as the rates of response to, and recovery from, environmental perturbations [5]. Furthermore, an animal’s response can vary in totally different environments. Traditionally, the focus of livestock breeding goals has been on traits that are directly related to production performance. Actually, the increasingly wide variety of environmental conditions in which livestock is required to perform is rapidly moving attention to robustness traits [6]. Rearing in increasingly heterogeneous environmental conditions increases the need of selecting resilient animals. In these conditions, animals can be exposed to different types of challenges or disturbances such as nutritional availability, thermal stress, disease pressure, etc. [5]. Under real productive rearing conditions, challenge events are often unrecorded and from unknown source, and in the past, the amount of frequently recorded data was not sufficient to quantify resilience, given that it involves dynamic processes that are difficult to follow with few records, as discussed by Friggens et al. [5].

Repeated measurements over time have a high potential for the quantification of an animal’s ability to cope with environmental challenges [5]. Today, highly frequently recorded data are becoming increasingly available due to high-frequency recording systems such as automated monitoring technology or robot milking. The availability of a large volume of data enables to follow the whole process during which occurs the response of an animal to and the recovery from perturbations [5], but it also sets a challenge that involves dealing with a large amount of records. In general, the information recorded is related to productive performance (e.g. weights, milk production, feed intake, etc.) and, in some cases, also to climatic factors. However, little information (if any) is available for variables that can help identifying other environmental challenges such as problems related to health (e.g. pathogen load), nutritional conditions, the constraints imposed by the farming system, management, etc. However, the effect of environmental challenges on animal performance can be observed indirectly, through changes in variability patterns of repeated records of performance over time as shown by Nguyen-Ba et al. [7] for voluntary feed intake in pigs. In small ruminants, Colditz and Hine [1] and Friggens et al. [8] reported significant variation between animals in their response profiles to thermal and nutritional challenges, respectively. They observed that increased variation was related to the occurrence of an environmental challenge.

Several approaches have been proposed to identify variation in high-frequency datasets that may be related to the occurrence of environmental challenges. For example, Codrea et al. [9] presented a smoothing approach to detect deviations of production traits related to disturbances in the time-series of milk production. Berghof et al. [10] proposed resilience indicators based on standardized body weight deviations in layer chicken. Nguyen-Ba et al. [7] proposed a procedure to detect the impact of perturbations in growing pigs and quantify the response in feed intake in terms of resistance and resilience. Their method involved the estimation of a target trajectory of cumulative feed intake and, then the detection of the deviations from this target curve. In all these studies, challenges were artificially introduced and consequently, known and recorded. Poppe et al. [11] proposed three indicators based on fluctuation patterns in milk yield related to unknown disturbances. For these three indicators, it is necessary to estimate a lactation curve for each animal as a first step. All these studies show that there is increased variability related to environmental challenges but there is no standard and widely applicable method to detect perturbations in rearing conditions in which only records of the traits of interest are available with very little (if any) information related to environmental conditions.

The objective of this study was two-fold: (1) to present a simple and practical data-driven approach to estimate the probability that, at a given date, an unrecorded environmental challenge occurred, using a mixture model of phenotypic variances, and (2) to evaluate the genetic determinism of resilience to these events, using these probabilities as covariates in a reaction norm model. In this study, we analyzed an ovine dataset but it is important to note that the methods applied here are very general and can be used for other species and traits.

## Methods

We present a method that consists first, in inferring the existence of highly variable days (where high variation among individuals in phenotype is a putative indicator of environmental challenge) via mixture models applied to frequently recorded phenotypic measures, and second, in using the inferred probabilities of the occurrence of an environmental challenge in a reaction norm model to evaluate the genetic determinism of resilience to these events. We illustrate the method by using an ovine dataset with daily feed intake (DFI) records.

### Finite mixture models and estimation of the probability of the occurrence of an unrecorded environmental challenge for each day

As we indicated previously, a simple indicator of an environmental challenge is an increased phenotypic variation due to more extreme individual responses to stress. According to McLachlan and Peel [12], in practice, there are cases where the population is a mixture of $n$ distinct groups that are known a priori to exist in some physical sense. Finite mixture models can be applied when there is group-structure in the data [13]. For instance, a source of heterogeneity can be age, sex, a disease (presence or absence), etc. [12]. Based on the same logic, we hypothesized that environmental challenges produce different reactions in different animals, leading to larger phenotypic variance as shown in previous studies (e.g. [1, 7, 8]). In fact, Scheffer et al. [14] refer to phenotypic variance as a “dynamic indicator of resilience”, since it can be useful to dynamically monitor changes in a system. Thus, we expect a dataset to contain a (at least) two-component normal mixture model: one for “normal” days, and another for “stressful” days with this high variability being related to the occurrence of an environmental challenge. However, the number of components is unclear and should be inferred from the data.

Following Chen et al. [15], the trait values related to the presence or absence of a disturbance are distributed as $N({\mu }_{1}, {\sigma }_{1}^{2})$ and $N({\mu }_{2}, {\sigma }_{2}^{2})$, respectively, and thus the quantitative trait under study in the population has a two-component normal mixture distribution:

$$Var\left(y\right)=\sum_{i=1}^{2}{\alpha }_{i}N({\mu }_{i}, {\sigma }_{i}^{2}),$$

where ${\alpha }_{i}$ are the mixing proportions (non-negative and summing to 1) and $N({\mu }_{i}, {\sigma }_{i}^{2})$ are the component densities.

There are different approaches to estimate mixture distributions. The most commonly used approach fits mixture models by maximum likelihood estimation via the expectation–maximization (EM) algorithm of Dempster el al. [12, 16], which allows to estimate the distribution component parameters, mixing proportions and posterior component membership probabilities. These are probabilities that a given day belongs to the first or second component of the mixture. These probabilities can be used for two purposes, either for clustering (“normal” or “stressful” days) or directly as an environmental descriptor continuous variable to characterize the environmental gradient. A problem in clustering is the choice of an arbitrary cut-off, with loss of information, in particular, if the two components of the mixture overlap. The mixture model needs data, so it is expected to be accurate with frequently recorded data (e.g. daily) but not with sporadically recorded data (e.g. monthly). Preliminary tests with monthly milk recordings in dairy sheep did not show the existence of more than one component in the mixture (results not shown), and we attribute this to the lack of more frequent measures. Moreover, the method requires homogeneity of animals within groups, to avoid variability originating from differences in age, for example, i.e. in that case, lambs and adults should be analyzed separately.

Thus, our method is as follows:

1. Fit a mixture model to data. On output, there may be two (or more) components. The component related to larger variance values is associated to stress or challenge.
2. Include the probability of belonging to the “stressful” component as a covariate in a reaction norm animal model.

This approach is simple and practical because it enables analysis of all the phenotypic data together from all individuals, and estimation of the probability that, at a given date, an unrecorded environmental challenge occurred based on the variability observed for each day. There is no need for an initial step to estimate, first, an observed or a target curve (e.g. a growth or milk yield curve), with all the parameters that this implies, and, second, the deviations from the curve as indicators of environmental challenges. Thus, instead of measuring deviations within individuals across time, we measure deviations across individuals within time. Moreover, this approach is sufficiently general to be applied to various types of datasets coming from different species and with records for different kinds of traits, as it only focuses on daily (or other time frame) variability.

### Animals and phenotypes

The present study was carried out in agreement with the French National Regulations for humane care and use of animals for research purposes (Décret 2013/118). Animals were bred at the experimental INRAE Farm (La Sapinière, Osmoy, France) which has the experimental approval C18‐174‐01.

Data are fully described in [17, 18], and here, we only summarize the main aspects. Data for the analysis were from 951 Romane male lambs that are evaluated as part of the national Romane breeding scheme. Over an 8-year period (from 2009 to 2016), each year, a cohort of 119 lambs on average (ranging from 92 to 149 depending on the year) was continuously phenotyped during eight weeks for feeding behavior. They were housed in the same experimental barn during the 8-week period. Although animals were reared indoors, environmental variables such as temperature or humidity were neither recorded nor regulated, and rearing conditions were similar across years. Each year, after weaning, all the lambs of approximately the same age (on average 70 days old, ranging from 60 to 90 days) were grouped in five to eight pens with a mean of 20 animals per pen (ranging from 13 to 27). Each pen was equipped with an automatic concentrate feeder (ACF). A 14-day period of adaptation to the new environment was necessary, followed by an 8-week period during which the animals were tested in winter (from December to February).

Lambs were fed low-energy concentrated pellets ad libitum. Each year, feed intake was recorded continuously during the 8-week period in winter. For each record (hereafter called “a visit”), the animal was identified by the ACF and the quantity of concentrate consumed and the duration of the visit were recorded. More details regarding the low-energy concentrated diet and how the ACF operates are in [17, 18].

In total, 775,580 visit records were available for all eight periods, with an average of 14.56 visits per day per animal. All visit records within each day for each animal were summed up to obtain the daily feed intake (DFI), resulting in 51,832 DFI records available for all the animals during the eight years. As the data were recorded in growing animals, DFI (and its variance due to a scale effect) tends to increase as the animal gets larger over time. In order to take this into account, we used the (natural logarithm of) daily coefficient of variation (CV) instead of the daily variance to assess variability. This represents a total of 438 days across the eight years, i.e. 55 days per year on average. For the genetic analysis, the pedigree consisted of 5114 animals, i.e. the 951 tested lambs and their ancestors.

### Fitting a finite mixture model and estimation of the probabilities of the occurrence of an unrecorded environmental challenge in a Romane lamb population

We used the *normalmixEM* function in the R library *mixtools* [19] to implement the Gaussian mixture model to the data consisting of 438 values of log(CV) registered for each day during the eight years. The mixture fitting procedure involves the expectation–maximization (EM) algorithm [12]. The mean and variance of both components were initially unconstrained. Although we had some a priori information to fit a mixture of two components, we used a parametric bootstrap to confirm the number of components. This procedure tests sequentially the null hypothesis of a $k$-component mixture Gaussian distribution [12, 19]. In this way, we were able to confirm that a mixture of two normal components was appropriate to model the data.

In addition, for each day (within each year), we computed the posterior probabilities of pertaining to the first or the second component of the mixture distribution. Days with a high probability of pertaining to the first component were “low CV days”, and those with a high probability of pertaining to the second component “high CV days”. Days with a high probability of having a high CV showed increased variability, which is probably related to the occurrence of an environmental challenge. The probabilities of pertaining to the second component were taken as a reference and used in the genetic analysis described below. In summary, these probabilities quantify the possibility that, at a given date, an unrecorded environmental challenge occurred.

These probabilities can be used directly (as a continuous variable) in a genetic model to describe the environment without the need to assign each of the days to a discrete (categorical) variable. These probabilities permit the description of the environment through a gradient going from a non-challenging ($p$ = 0) to a challenging environment ($p$ = 1), and the intensity of the challenge can be quantified through the probability value. Moreover, each individual has one DFI record per day and there is one probability $p$ per day, and these probabilities vary between days. For these two reasons, it is convenient to use a reaction norm model that includes these probabilities as an environmental descriptor as presented below.

De Jong [20] defined the reaction norm as the total pattern of expression of a character along a continuous gradient given by an environmental descriptor. In their paper, Calus and Veerkamp [21] discuss that environmental descriptors should (i) reflect management and environment, (ii) be obtained from available data, and (iii) be continuous rather than categorical. The estimated probabilities that, at a given date, an unrecorded environmental challenge occurred, meet these conditions.

### Genetic analysis

Phenotypes (DFI) were analyzed using a linear reaction norm animal model (RNAM) including the estimated probabilities that, at a given date, an unrecorded environmental challenge occurred, to evaluate the genetic determinism of resilience to unrecorded environmental challenges. Here, an approximately linear response (i.e. there is no intermediate optimum or change point) was assumed. However, the probabilities can be used in more complex regressions (e.g. quadratic or piece-wise) if required. The model was:

$${y}_{ijk}={yearACF}_{i}+{{b}_{1}day}_{j}+{a}_{0,k}+{a}_{1,k}*{p}_{j}+{pe}_{0,k}+ {pe}_{1,k}*{p}_{j}+{e}_{ijk},$$

where ${y}_{ijk}$ is the observation of DFI (in kg) in yearACF $i$, on day $j$ for animal $k$. Preliminary analyses were performed by Marie-Etancelin et al. [18] and Tortereau et al. [17] to determine the fixed effects that should be taken into account in the genetic analyses. Only two fixed effects were significant: year (eight levels) and ACF device (up to seven levels per year). These were combined into one term (${yearACF}_{i}$, $i\hspace{0.17em}$= 1 to 44) in the model. The second term corresponds to a regression on day $j$ to take the effect of growing over the test period on DFI into account; ${a}_{0,k}$ is the breeding value (BV) for level (or intercept) of DFI and corresponds to the classical BV for the performance potential of animal $k$ (it is environment-independent); ${a}_{1,k}$ is the BV for slope (environmental sensitivity) of DFI for animal $k$, ${p}_{j}$ is the probability that on day $j$ an unrecorded environmental challenge occurred; ${pe}_{0,k}$ is the permanent environmental effect of animal $k$ (intercept), ${pe}_{1,k}$ is the permanent environmental effect of animal $k$ for ${p}_{j}$ (slope); and ${e}_{ijk}$ is the residual. A slope of ${a}_{1,k }=0$ means that the animal is not sensitive to stress and a ${a}_{1,k}$ higher or lower than 0 means that the animal takes more or less food in stressful environments, respectively.

The ${a}_{0}$ and ${a}_{1}$ are assumed to follow a bivariate distribution with $$Var\left(\begin{array}{c}{a}_{0}\\ {a}_{1}\end{array}\right)=\left(\begin{array}{cc}{\sigma }_{a0}^{2}& {\sigma }_{a0,a1}\\ {\sigma }_{a0,a1}& {\sigma }_{a1}^{2}\end{array}\right)$$, and ${pe}_{0}$ and ${pe}_{1}$ are assumed to follow a bivariate distribution with $$Var\left(\begin{array}{c}{pe}_{0}\\ {pe}_{1}\end{array}\right)=\left(\begin{array}{cc}{\sigma }_{pe0}^{2}& {\sigma }_{pe0,pe1}\\ {\sigma }_{pe0,pe1}& {\sigma }_{pe1}^{2}\end{array}\right)$$. Residual variances were assumed to be homogeneous. A RNAM with heterogeneous residual variance was fitted to assess the sensitivity of the results to how the residual variance is modelled. In this case, one residual variance was set for normal days and one for highly variable days. Nine values of $p$, ranging from 0.10 to 0.90 were set as cutting points to differentiate between normal days and highly variable days (see Additional file 1: Table S1).

For comparison, another animal model (AM) was fitted without the reaction norm terms, i.e. without ${a}_{1,k}*{p}_{j}$ and ${pe}_{1,k}*{p}_{j}$.

### Estimation of variance components and comparison of models

(Co)variance components were estimated using Gibbs sampling and REML with the GIBBSF90 and AIREMLF90 software (available at http://nce.ads.uga.edu/wiki/), respectively [22]. For Gibbs sampling, 200,000 iterations were run, with a burn-in of 10,000 initial iterations and a sample interval of 10. Posterior means and posterior standard deviations (SD) were calculated. For REML, the asymptotic standard error for the genetic correlation was computed following Houle and Meyer [23], as implemented in AIREMLF90. For this purpose, first we ran EM-REML for all the initial iterations and then switched to AI in the final iteration because the EM-REML algorithm is much more stable than the AI algorithm and is very robust to poor initial estimates and can thus provide a good starting point for the AI algorithm [24].

A likelihood ratio test was performed from the REML results to assess goodness-of-fit and to compare the RNAM and traditional AM. ${\chi }^{2}$ values were calculated as ${\chi }^{2}=-2log{L}_{AM}+2log{L}_{RNAM}$, with the first and second terms being the AM and the RNAM likelihood, respectively. *P*-values of the Chi-squared tests were obtained from a mixture of Chi-squared distributions with two and four degrees of freedom [25, 26].

For a given level of the covariate $p$, the total genetic variance is equal to ${\sigma }_{a0}^{2}+2p{\sigma }_{a0,a1}+{p}^{2}{\sigma }_{a1}^{2}$, similarly for the variance due to the permanent environment, and from here it is possible to obtain values of heritability that change across conditions (i.e. from $p\hspace{0.17em}= 0 {\text{to}} 1$).

The genetic correlation between breeding value in a non-challenging environment ($p=0$) and breeding value at a given probability of the occurrence of an environmental challenge ($p$) was calculated as:

$$Cor({a}_{0},{a}_{p})=\frac{Cov({a}_{0},{a}_{p})}{\sqrt{Var({a}_{0})Var({a}_{p})}}=\frac{{\sigma }_{a0}^{2}+{\sigma }_{a0,a1}*p}{\sqrt{{\sigma }_{a0}^{2}\left({\sigma }_{a0}^{2}+{p}^{2}{\sigma }_{a1}^{2}+2p{\sigma }_{a0,a1}\right)}}.$$

## Results

### Fitting a finite mixture model and estimation of the probabilities of the occurrence of an unrecorded environmental challenge in a Romane lamb population

**Fig. 1:**
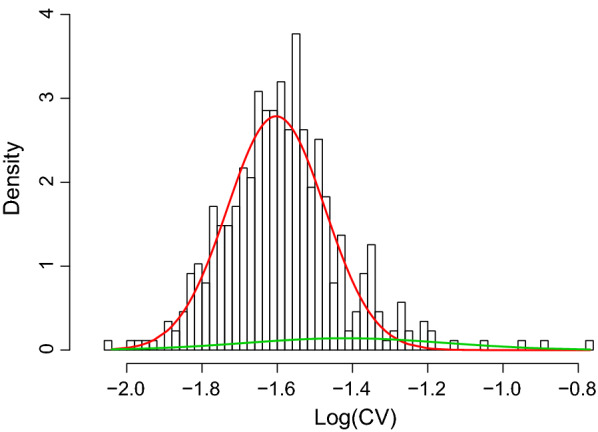
Plot of the log-transformed coefficient of variation (CV) of daily feed intake (DFI) data of the fitted two-component (red and green) normal mixture model

The histogram in Fig. 1 shows, on the right side, a subgroup of days that appear to have values of the natural logarithm of the CV of DFI that are higher than most the records on the left (higher than − 1.4). This was an initial hint that there could be two different groups of days regarding the variability of the DFI records. This was confirmed through parametric bootstrap [19]. Figure 1 also shows the density of the two-component normal mixture fitted on the log(CV) of DFI. The two components fitted are heteroscedastic and have different means. The first component (red in Fig. 1) has a mean of − 1.60 and a SD 0.13, and the second component (green in Fig. 1), a mean of − 1.42 and a SD 0.27.

**Fig. 2:**
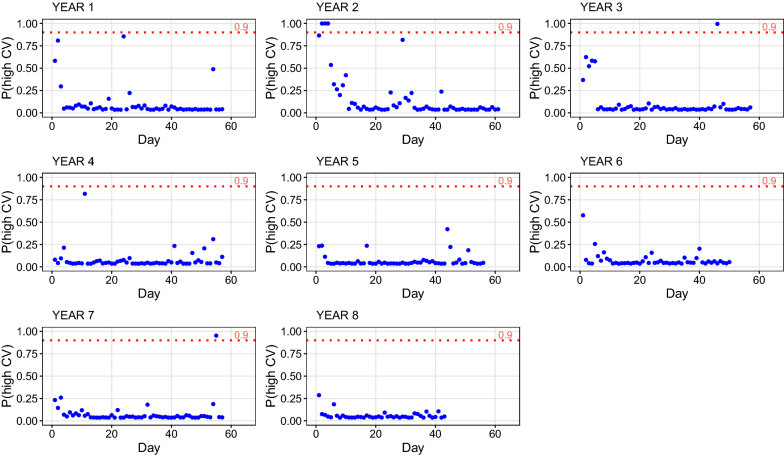
Probabilities of showing a high coefficient of variation (CV) related to the occurrence of an environmental challenge for each day. These values correspond to the probability of pertaining to the second component of the mixture of the two Gaussian distributions

The EM algorithm for mixture models allows to estimate the probability that each day belongs to the second component of the mixture (the probability of being a “high CV day”). These probabilities are shown in Fig. 2. The mean of these probabilities was 0.09, which indicates that for most of the days the probability of the occurrence of an environmental challenge was low. In Fig. 2, some years (2, 3, and 7) had days with a probability of the occurrence of an environmental challenge higher than 0.90. Furthermore, only 3.88% of the days across different years were found to have probabilities of being a challenge day higher than 0.5. Most days showed a probability lower than 0.25.

We carried out a posterior analysis that was focused on the days with a high estimated probability $p$ of the occurrence of an environmental challenge. Our aim was to check if these days corresponded to known disturbances on the experimental farm. We confirmed that some of the days with a high value of $p$ (high probability of being stressful) were associated to changes in management, weighing of the animals, mulching, or fixing (repairing) some of the ACF. However, for some other days with a high $p$, there was no explanation, and we assume that these corresponded to days with unrecorded environmental challenges.

### Genetic analysis

Table 1 shows the estimates of the variance components for DFI using the RNAM and AM models. They were not sensitive to how the residual variance was modelled. Additional file 1: Table S1 contains the estimates in the case of two residual variance components depending on $p$ and shows that the results are very similar to the single residual variance case that we describe here. The genetic correlation between the level and the slope of the reaction norm was − 0.46 ± 0.21, which shows that level is antagonistic to environmental sensitivity. A hypothetical selection for increased DFI under no stressful conditions (which is generally the case) would result in animals with decreased DFI in stressful conditions. On the contrary, a hypothetical selection for decreased DFI would result in animals with increased DFI in stressful conditions.

**Table 1:** Parameter estimates obtained using the animal model (AM) and the reaction norm animal model (RNAM)

Parameter	Model	
	AM	RNAM
${\sigma }_{a0}^{2}$	0.014	0.016
${\sigma }_{a1}^{2}$	–	0.038
${\sigma }_{{a}0,{a}1}$	–	− 0.011
${\sigma }_{{pe0}}^{2}$	0.028	0.034
${\sigma }_{{pe1}}^{2}$	–	0.104
${\sigma }_{{pe0},{pe1}}$	–	− 0.031
${\sigma }_{\mathrm{e}}^{2}$	0.123	0.120
Genetic correlation	–	− 0.460 ± 0.210

${\sigma }_{a0}^{2}=$ additive genetic variance for level, ${\sigma }_{a1}^{2}=$ additive genetic variance for slope, ${\sigma }_{{a0},{a1} }=$ additive genetic covariance between level and slope, ${\sigma }_{{pe0}}^{2}=$ permanent environmental variance for level, ${\sigma }_{{pe1}}^{2}=$ permanent environmental variance for slope, ${\sigma }_{{pe0},{pe1} }=$ permanent environmental covariance between level and slope, ${\sigma }_{\mathrm{e}}^{2}=$ residual variance

Goodness-of-fit of the models was assessed by performing a likelihood ratio test (Table 2). Model RNAM fitted the data much better than AM. This shows that the log(CV) is a valid indicator for the genetic analysis and may be of value to select for resilience.

**Table 2:** Goodness of fit, likelihood ratio test of models AM and RNAM

−2 log likelihood		Likelihood ratio test	
AM	RNAM	${\chi }^{2}$	p-value
41219.52	40685.54	533.98	1.50*10^–114^

**Fig. 3:**
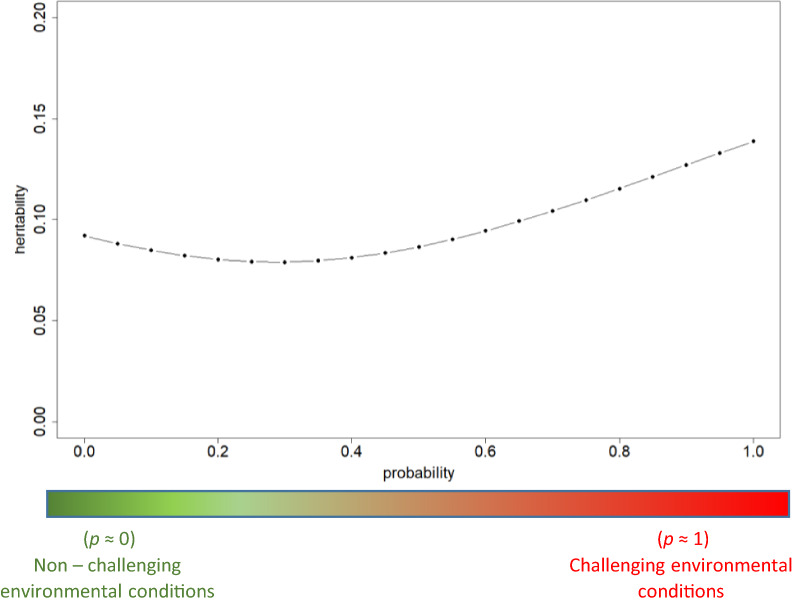
Heritability estimates of daily feed intake (DFI) for different probabilities of the occurrence of an environmental challenge. $p$ is the environmental descriptor with $p$ = 0 indicating non-challenging environmental conditions and  $p$ = 1 indicating highly challenging conditions

Figure 3 shows the heritability estimates for DFI for different probabilities of the occurrence of an environmental challenge. The interquartile range of its sampling distribution is shown in Additional file 2: Figure S1) and the standard error (SE) of the curve is roughly 0.04 along its trajectory. The values ranged from 0.08 (for $p$ = 0.30) to 0.14 (for $p$ = 1). Heritability for DFI decreased from $p$ = 0 to 0.30, and then increased for higher values of $p$. This behavior was comparable to that reported by Ravagnolo and Misztal [27] and Kolmodin et al. [28] in dairy cattle. In both studies, the genetic parameters changed across different environments. Marie-Etancelin et al. [18] reported a higher heritability for average DFI for the same ovine population analyzed in this study. Heritability of DFI is lower than that of average DFI because the latter contains less noise, as it is averaged over time.

**Fig. 4:**
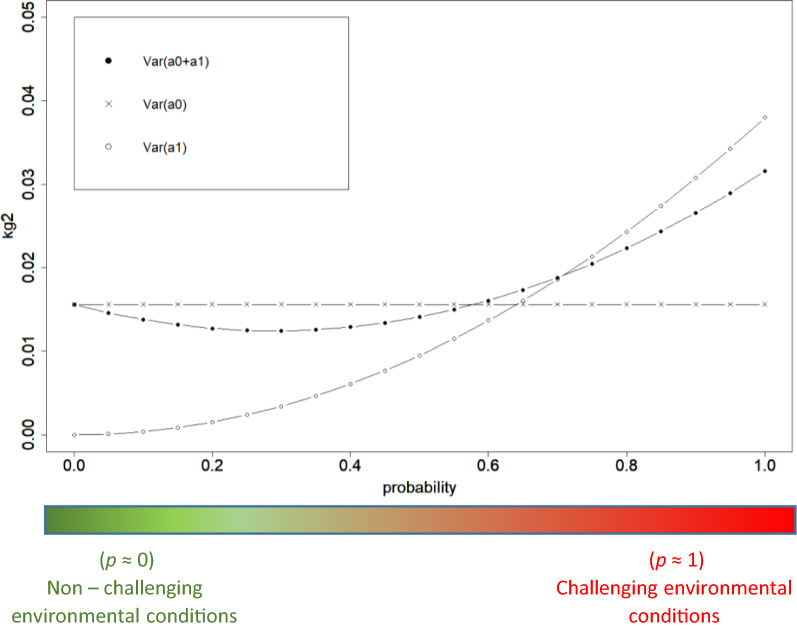
Level (${a}_{0}$), environmental sensitivity (${a}_{1}$) and total additive (${a}_{0}+{a}_{1}$) variances for daily feed intake (DFI) for different probabilities of the occurrence of an environmental challenge. $p$ is the environmental descriptor with  $p$ = 0 indicating non-challenging environmental conditions and  $p$ = 1 indicating highly challenging conditions

Figure 4 shows the estimates of level (${a}_{0}$), environmental sensitivity (${a}_{1}$) and total additive (${a}_{0}+p\times {a}_{1}$) variances for DFI for increasing probabilities of the occurrence of an environmental challenge (going from non-challenging environmental conditions to more challenging ones). The interquartile ranges of their sampling distributions are shown in Additional file 3: Figures S2–4. The SE for level variance, environmental sensitivity variance and total additive variance are lower than 0.006 along the trajectories. Under non-challenging environmental conditions ($p$ = 0), the additive variance for environmental sensitivity is 0 and starts to increase for higher values of $p$ (more challenging environmental conditions), reaching its maximum value (0.04 kg^2^) when $p$ = 1. As there is a negative covariance between level and slope, the total additive variance first decreases slightly as $p$ increases and then, for higher values of $p$, it starts to increase.

**Fig. 5:**
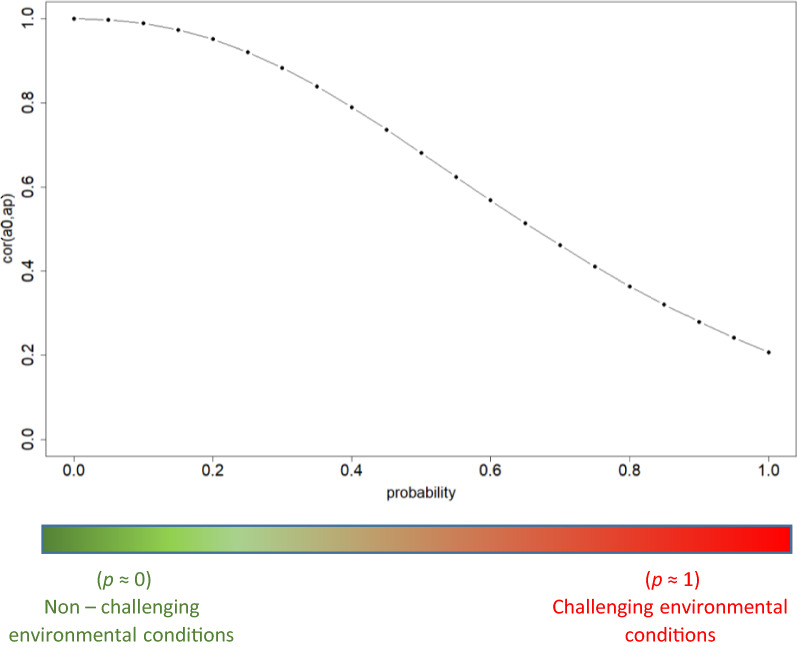
Genetic correlation between breeding values in a non-challenging environment ($p$ = 0) and breeding value at a given probability of the occurrence of an environmental challenge ($p$)

Figure 5 shows the genetic correlation between EBV in a non-challenging environment ($p$ = 0) and breeding value at a given probability of the occurrence of an environmental challenge ($p$). For low values of $p$ from 0 to 0.15, i.e. non-challenging environmental conditions, the correlation is close to 1 (from 0.97 to 1). For values of $p$ higher than 0.15, the correlation starts to decrease and reaches its minimum value (0.21) for $p$ = 1. Low genetic correlations between challenging and non-challenging environments indicate reranking of individuals in different environmental conditions. It also implies that selection under completely non-challenging conditions is ineffective for the expression of the trait in completely challenging conditions.

**Fig. 6:**
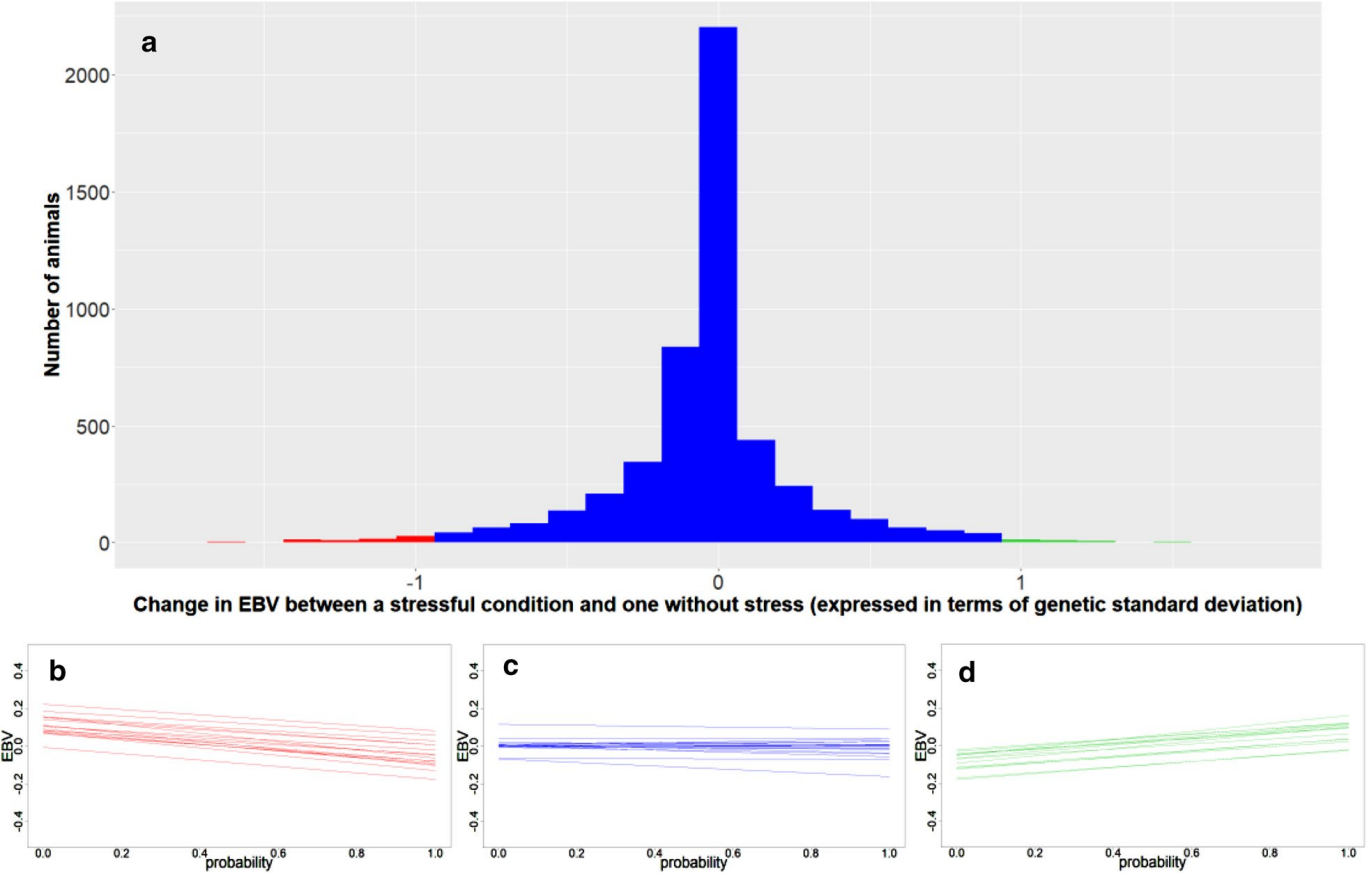
**a** Change in EBV between a non-challenging environmental condition ($p$ = 0) and an extremely challenging condition ($p$ = 1) expressed in terms of genetic standard deviation. Three groups were identified according to the pattern of reaction to environmental challenging conditions and are indicated with different colors on the histogram (red: animals with EBV that decrease for higher values of $p$; blue: animals with EBV that tend to remain approximately constant for higher values of $p$, within − 1 and + 1 genetic standard deviations; green: animals with EBV that increase for higher values of $p$). Twenty animals were randomly sampled from each group, and panels (**b**–**d**) show the reaction norms for daily feed intake (DFI) for each of the three groups

Figure 6 shows the change in EBV between non-challenging ($p$ = 0) and challenging environmental conditions ($p$ = 1) expressed in terms of genetic standard deviation. Three groups of animals were identified according to the pattern of change. The majority of the individuals (over 95%) were within the second group (in blue in Fig. 6). Figure 6b–d show that each group had a different pattern of response to increasing challenging environmental conditions. In the first group (red), the EBV for DFI decreased as $p$ increased, whereas the opposite occurred in the third group (green). Within each of these two groups, there were also significant differences in environmental sensibility (slope). Animals with the steepest slopes were more sensitive to environmental challenges, consequently less resilient. The second group (blue) includes animals that show an intermediate response in terms of the change in EBV according to the value of $p$. In this group, we can find animals with a positive or negative slope (or even close to zero) but changes in terms of EBV are within − 1 and + 1 genetic standard deviation. In other words, we can say that the second group is composed of animals that were less environmentally sensitive (with a less steep slope compared to the other groups). However, due to the genetic correlation between the level and the slope, hypothetical selection for increased DFI would shift the distribution to the left, whereas hypothetical selection for decreased DFI would shift the distribution to the right.

Figure 6 also shows that there is a reordering of genotype ranks and variation in EBV of the animals along the environmental gradient (given by the probability of the occurrence of an environmental challenge). This, combined to the results in Fig. 5 with low genetic correlations between extreme environmental conditions, illustrates the existence of G $\times$ E interaction [29].

## Discussion

### Finite mixture models and estimation of the probability of the occurrence of an unrecorded environmental challenge on each day

Previous studies, which were based on artificially introduced disturbances (e.g. [1, 7, 8]), showed that there is increased variability in the presence of environmental challenges. In this study, we presented a simple and practical approach to estimate the probability that, at a given date, an unrecorded environmental challenge occurred. This is a data-driven approach, since the estimations are based only on phenotypic data with no need of extra information as for example climatic information. Compared to other methods, our approach does not need an initial step to estimate an observed or a target curve (with all the parameters that this implies), and the deviations from the curve to detect the occurrence of environmental challenges. Furthermore, our approach can be applied to various types of datasets from different species and with records on different kinds of traits because it focuses only on daily (or other time frame) variability, without the need to assume any kind of curve or function for the trait or traits analyzed. Consequently, the method is widely applicable. However, the method requires frequently recorded (daily or similar) data and homogeneity of animals within groups – for instance, lambs and adults should be analyzed separately. Daily recording is not frequent but there are more and more automatic measuring devices such as milking robots that provide records that can be used. As described by Friggens et al. [5], technology for the measurement of milk yield and body weight at each milking is commercially available and used routinely in some milking systems. Systems that automatically weigh animals, either directly or using image analysis of body shape, have been developed. Imaging technology has recently been commercialized for measuring body condition [30]. There are also a number of monitoring technologies for measuring animal behavior (accelerometers, position tracking, video), and an increasing number of sensors for measuring biomarkers of different aspects of health status in milk, in exhaled breath, and in situ with under-skin sensors or boluses in the rumen [5]. Today, dealing with large amounts of records produced by all these monitoring technologies is a challenge. For very large datasets (e.g. as in dairy cattle national evaluations), genetic connections (i.e. through sires) may reveal classes of variances as in our study.

In this paper, we illustrate the analysis of data and the modeling procedure proposed by using a dataset with DFI records from Romane lambs across eight years. Our results indicate that this approach is promising and appears to be viable to identify unrecorded environmental challenge events, which is useful when selecting resilient animals and only productive data are available. Although our illustration uses an ovine dataset with DFI records, the proposed approach can be applied to a wide variety of phenotypic records from different species and, given its simplicity, is useful when dealing with large quantities of data coming, for example, from high frequency recording systems such as automated monitoring technology. To our knowledge, there are no similar approaches proposed in the literature.

### Genetic analysis

Detection of environmental challenges that affect the entire population (or contemporary group) can provide a unique opportunity to select resilient animals (or less environmentally sensible) to such events since comparable individuals are exposed to the same environmental conditions. In this study, we evaluated the genetic determinism of resilience to these events using the probabilities of the occurrence of an environmental challenge as an environmental descriptor in a reaction norm animal model. We were able to estimate breeding values for level and environmental sensitivity of DFI for each animal and our results indicate that there is genetic variation in environmental sensitivity. This kind of analysis enables the identification of animals that combine both, high production potential with resilience to environmental challenges. Even if the environmental challenges are unknown and correspond to infrequent combinations of infrequent factors, the animals resilient to this kind of stress should be resilient to different stressors (heat, changes in management, pathogen load, etc.) which is desirable. In a way, we can think of these stresses as an “infinitesimal”–sum of different small factors.

Animal breeding practices have become part of the debate since it is now recognized that animals selected for high production efficiency are more at risk for behavioral, physiological and immunological problems and are generally less resilient [31–33]. Consequently, it is important to include resilience or robustness within the breeding goals, but resilience is not (yet) included in breeding goals of livestock [2].

As discussed by Knap [6], one feasible way of breeding for improved animal robustness (or resilience) is to estimate breeding values for the environmental sensitivity of the genetic potential for performance through the use of reaction norm analysis. Simms [34] proposed to use the slope of the reaction norm as a resilience (to which they refer as tolerance) indicator, which was later used in several studies (e.g. [2, 35]). Berghof et al. [2] claim that the slope of the reaction norm indicates resilience towards macro-environmental disturbances (i.e. stressors that affect the whole population, e.g. temperature). It is within this context that we propose a method to identify environmental challenges and to estimate their probability of occurrence and introduce an alternative environmental descriptor that takes unobserved macro-environmental disturbances to which animals could have been exposed into account.

In this study, we obtained a negative genetic correlation between level of DFI and sensitivity to inferred environmental challenge, which indicates that a hypothetical selection for increased DFI in non-stressed environments would result in animals that are suboptimal for DFI in stressed environments. In the opposite scenario, a hypothetical selection for decreased DFI would result in animals with increased DFI in stressful conditions. In both cases, slopes will become steeper (negative or positive, respectively), showing an increase in environmental sensitivity. Negative correlations between level and slope result from resource allocation patterns as described by Beilharz et al. [36]. Resource-demanding physiological processes show trade-offs that result from limits in resource availability. In this way, animals that are genetically driven to produce at high levels will probably reallocate resources away from processes related to resilience. A reordering of individual ranks was observed along the environmental gradient as well as a low genetic correlation between extreme environmental conditions. Animals that were good (top of the rank) at $p$ = 0 (no challenge) tended to be poor (bottom of the rank) at $p$ = 1 (challenge). These observations confirmed the existence of a G $\times$ E interaction and indicate that the best genotype in one environmental condition is not the best in another one [37]. Knowledge of the existence of G $\times$ E interaction is important in terms of selection decisions.

Friggens et al. [5] proposed a categorization of animals into generalist and specialist individuals based on the slope of the individual random regression. On the one hand, generalist individuals are animals with a slope relatively close to zero, with a constant performance across different environmental conditions. On the other hand, specialist individuals are animals with steep slopes (both positive or negative), which show a significantly better performance in extreme environmental conditions (either challenging or non-challenging). However, it is difficult to directly select for generalist animals as this is an intermediate optimum. A possibility would be to select based, not on the reaction norm model (which may indicate the possible existence of resource allocation conflicts), but on the genetic basis of variation itself [38].

Previous studies have addressed the issue of resilience with different approaches. For example, some studies analyze the genetic potential of the variance of deviations as an indicator for resilience in dairy milk yield and DFI in pigs [11, 39, 40]. Berghof et al. [10] proposed skewness and autocorrelation of body weight deviations in layer chickens. Here, we propose an alternative approach and implemented it on an ovine population. As far as we know, such research on small ruminants is rare mostly because of the lack of high frequency records, which is a key element to work on resilience. However, resilience in small ruminants is important as they are commonly exposed to heterogeneous and changing conditions with little control of environmental factors compared to other livestock species, in which production systems are more controlled such as for monogastrics. Given these conditions, it is necessary to select animals that can maintain their production performance (or modifying it as little as possible) under this heterogeneous environment. This is the reason why resilience to changing environmental factors is of interest when selecting individuals. However, it is also necessary to account for the fact that this is a complex trait composed of multiple components, including dynamic elements such as the rates of response to, and recovery from environmental perturbation, which can lead to different responses in different environments.

## Conclusions

Today, very frequently recorded data are becoming increasingly available, due to the availability of high-frequency recording systems such as automated monitoring technologies. The method that we propose consists, first, in inferring the existence of highly variable days via mixture models applied to frequent phenotypic records and second, in using the inferred probabilities of a mixture in a norm reaction model. The approach is simple and practical and can be widely used for different species and traits. It enables the estimation of the probability of the occurrence of an environmental challenge for each day, and this variable proved to be informative and useful to be included in genetic analyses as an environmental descriptor to select resilient animals. We estimated breeding values for environmental sensitivity of the genetic potential for DFI through the use of reaction norm analysis. The level and slope were negatively correlated, which indicates that a hypothetical selection for increased DFI may not be optimal depending on the presence or absence of stress. A reordering of individual ranks was observed along the environmental gradient as well as low genetic correlations between extreme environmental conditions. These results confirmed the existence of G $\times$ E interaction and indicate that the best genotype in one environmental condition is not the best in another one.

## Supplementary Information

**Additional file 1: Table S1.** Parameter estimates obtained using the reaction norm animal model (RNAM) with homogeneous residual variance (column in grey) and heterogeneous residual variances (last nine columns). One residual variance was set for normal days and one for highly variable days. Nine values of *p*, going from 0.10 to 0.90 were set as cutting points to differentiate between normal days and highly variable days.

**Additional file 2: Figure S1.** Heritability estimates for daily feed intake (DFI) for different probabilities of the occurrence of an environmental challenge ($p$ is the environmental descriptor with  $p$ = 0 indicating non-challenging environmental conditions and  $p$ = 1 indicating highly challenging conditions). The interquartile range is shown in grey.

**Additional file 3: Figure S2.** Total additive (${a}_{0}+{a}_{1}$) variance for daily feed intake (DFI) for different probabilities of the occurrence of an environmental challenge. **Figure S3. **Level (${a}_{0}$) variance for daily feed intake (DFI) for different probabilities of the occurrence of an environmental challenge. **Figure S4.** Environmental sensitivity (${a}_{1}$), variance for daily feed intake (DFI) for different probabilities of the occurrence of an environmental challenge. In all cases $p$ is the environmental descriptor with  $p$ = 0 indicating non-challenging environmental conditions and  $p$ = 1 indicating highly challenging conditions. The interquartile range is shown in grey in all cases.

## Data Availability

The datasets analysed during the current study are available from the corresponding author on reasonable request.
