# Supplementary material for: Detection of unrecorded environmental challenges in high-frequency recorded traits, and genetic determinism of resilience to challenge, with an application on feed intake in lambs
Source: Genet Sel Evol. 2021 Jan 6;53:4. doi: 10.1186/s12711-020-00595-x (PMC7788967; doi:10.1186/s12711-020-00595-x)
Supplement: Supplementary file 1 — Additional file 1: Table S1. Parameter estimates obtained using the reaction norm animal model (RNAM) with homogeneous residual variance (column in grey) and heterogeneous residual variances (last nine columns). One residual variance was set for normal days and one for highly variable days. Nine values of p, going from 0.10 to 0.90 were set as cutting points to differentiate between normal days and highly variable days. [file 12711_2020_595_MOESM1_ESM.docx]

**Table S1.** Parameter estimates obtained using the reaction norm animal model (RNAM) with homogeneous residual variance (column in grey) and heterogeneous residual variances (last 9 columns). One residual variance was set for normal days and one for highly variable days. Nine values of *p*, going from (*p* >0.10 to *p* >0.90) were set as cutting points to differentiate between normal days and highly variable days.

|  | **Homogeneous residual variance** | **Heterogeneous residual variance.**  **2 classes according to different values of *p* (probability of having a high CV)** | | | | | | | | |
| --- | --- | --- | --- | --- | --- | --- | --- | --- | --- | --- |
|  |  | p >0.10 | p >0.20 | p >0.30 | p>0.40 | p>0.50 | p>0.60 | p>0.70 | p>0.80 | p>0.90 |
| $\sigma_{a0}^{2}$ | 0.016 | 0.01461 | 0.01455 | 0.01465 | 0.01464 | 0.01460 | 0.01500 | 0.0151 | 0.0151 | 0.0148 |
| $\sigma_{a1}^{2}$ | 0.038 | 0.0277 | 0.02769 | 0.02658 | 0.02647 | 0.02407 | 0.026024 | 0.02495 | 0.02495 | 0.0250 |
| $\sigma_{a0,a1}$ | -0.011 | -0.00938 | -0.00926 | -0.00923 | -0.00951 | -0.00925 | -0.01031 | -0.01028 | -0.01028 | -0.00928 |
| $\sigma_{pe0}^{2}$ | 0.034 | 0.03459 | 0.03436 | 0.034053 | 0.034037 | 0.03424 | 0.03395 | 0.03386 | 0.03386 | 0.03473 |
| $\sigma_{pe1}^{2}$ | 0.104 | 0.07996 | 0.07347 | 0.07738 | 0.07321 | 0.080931 | 0.08221 | 0.08379 | 0.08379 | 0.09954 |
| $\sigma_{pe0,pe1}$ | -0.031 | -0.03135 | -0.03012 | -0.02967 | -0.02950 | -0.03040 | -0.03064 | -0.03082 | -0.03082 | -0.03556 |
| $\sigma_{e}^{2}$ | 0.120 | 0.1566/ 0.1134 | 0.1704/ 0.1150 | 0.1685/ 0.1175 | 0.1815/ 0.1175 | 0.1751/ 0.1181 | 0.2073/ 0.1182 | 0.2181 / 0.1182 | 0.2181 / 0.1182 | 0.2927/ 0.1183 |
| -2 log likelihood | 40685.54 | 40339.28 | 40352.38 | 40538.19 | 40506.36 | 40564.482 | 40535.22 | 40529.92 | 40529.92 | 40514.70 |

$\sigma_{a0}^{2}=$ additive genetic variance for level , $\sigma_{a1}^{2}=$ additive genetic variance for slope, $\sigma_{a0,a1}=$ additive genetic covariance between level and slope, $\sigma_{pe0}^{2}=$ permanent environmental variance for level, $\sigma_{pe1}^{2}=$ permanent environmental variance for slope, $\sigma_{pe0,pe1}=$ permanent environmental covariance between level and slope, $\sigma_{e}^{2}=$ residual variance(s).
