# Supplementary material for: Detection of unrecorded environmental challenges in high-frequency recorded traits, and genetic determinism of resilience to challenge, with an application on feed intake in lambs
Source: Genet Sel Evol. 2021 Jan 6;53:4. doi: 10.1186/s12711-020-00595-x (PMC7788967; doi:10.1186/s12711-020-00595-x)
Supplement: Supplementary file 2 — Additional file 2: Figure S1. Heritability estimates for daily feed intake (DFI) for different probabilities of the occurrence of an environmental challenge ($p$p is the environmental descriptor with $p$p = 0 indicating non-challenging environmental conditions and $p$p = 1 indicating highly challenging conditions). The interquartile range is shown in grey. [file 12711_2020_595_MOESM2_ESM.pdf]

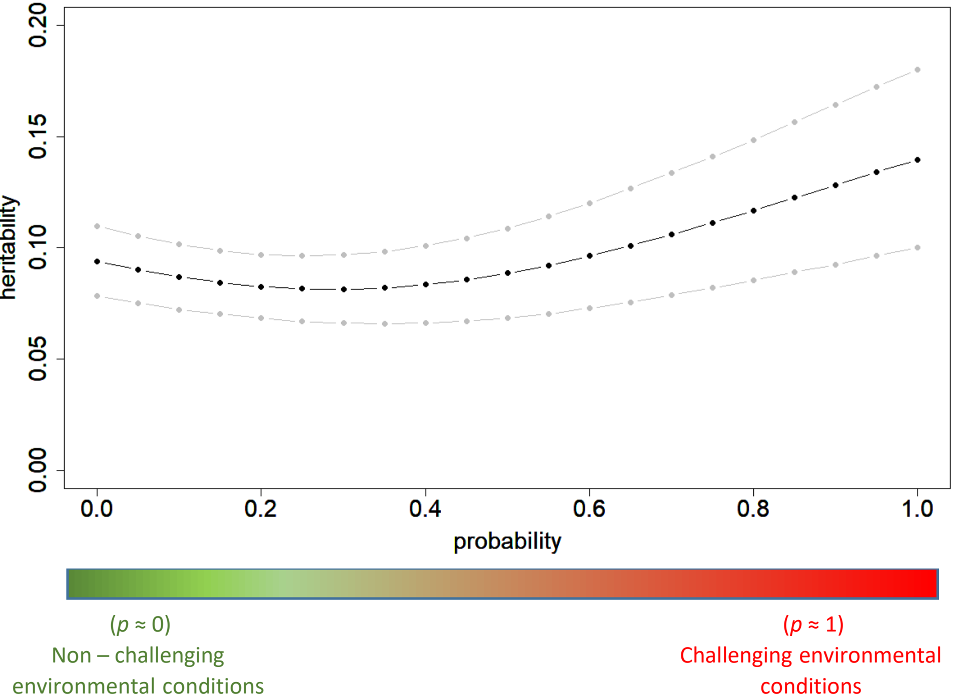
0.00
0.05
0.10
0.15
0.20
heritability
0.0
0.2
0.4
0.6
0.8
1.0
probability
($p \approx 0$)
Non – challenging
environmental conditions
($p \approx 1$)
Challenging environmental
conditions
