# Supplementary material for: Detection of unrecorded environmental challenges in high-frequency recorded traits, and genetic determinism of resilience to challenge, with an application on feed intake in lambs
Source: Genet Sel Evol. 2021 Jan 6;53:4. doi: 10.1186/s12711-020-00595-x (PMC7788967; doi:10.1186/s12711-020-00595-x)
Supplement: Supplementary file 3 — Additional file 3: Figure S2. Total additive (${a}_{0}+{a}_{1}$a0+a1) variance for daily feed intake (DFI) for different probabilities of the occurrence of an environmental challenge. Figure S3. Level (${a}_{0}$a0) variance for daily feed intake (DFI) for different probabilities of the occurrence of an environmental challenge. Figure S4. Environmental sensitivity (${a}_{1}$a1), variance for daily feed intake (DFI) for different probabilities of the occurrence of an environmental challenge. In all cases $p$p is the environmental descriptor with $p$p = 0 indicating non-challenging environmental conditions and [file 12711_2020_595_MOESM3_ESM.pdf]

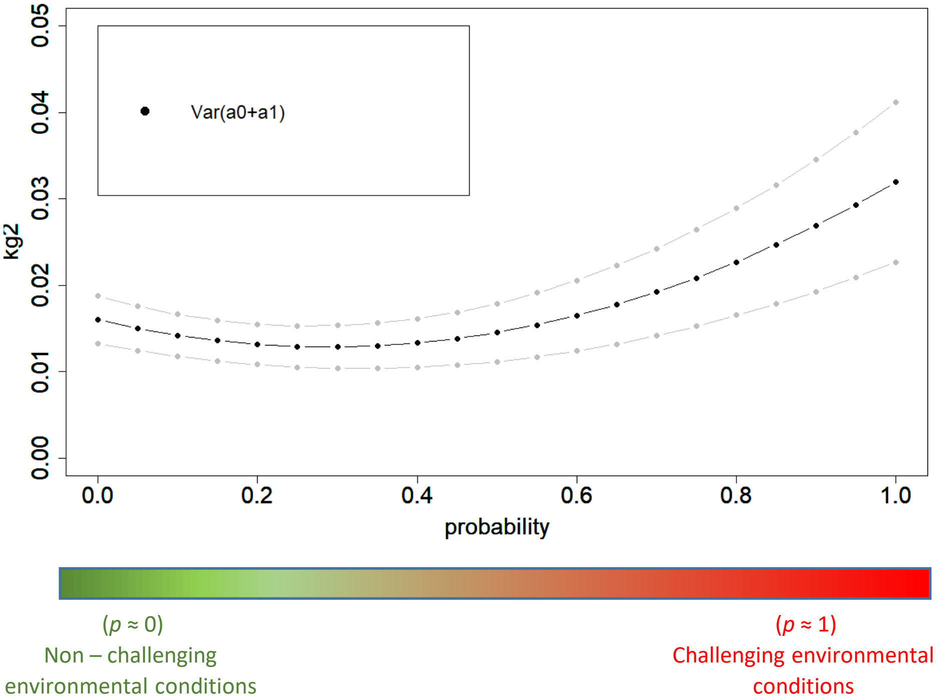

Var(a0+a1)
kg2
0.00
0.01
0.02
0.03
0.04
0.05
0.0
0.2
0.4
0.6
0.8
1.0
probability
(p ≈ 0)
Non – challenging environmental conditions
(p ≈ 1)
Challenging environmental conditions

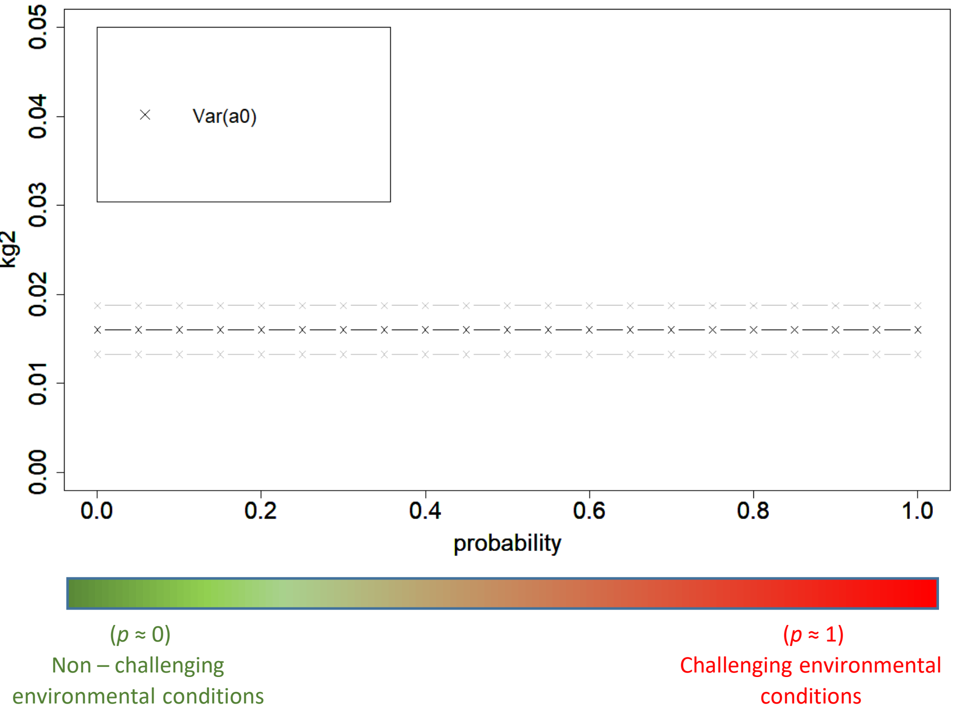

0.05
0.04
0.03
0.02
0.01
0.00
kg2
Var(a0)
0.0
0.2
0.4
0.6
0.8
1.0
probability
(p ≈ 0)
Non – challenging environmental conditions
(p ≈ 1)
Challenging environmental conditions

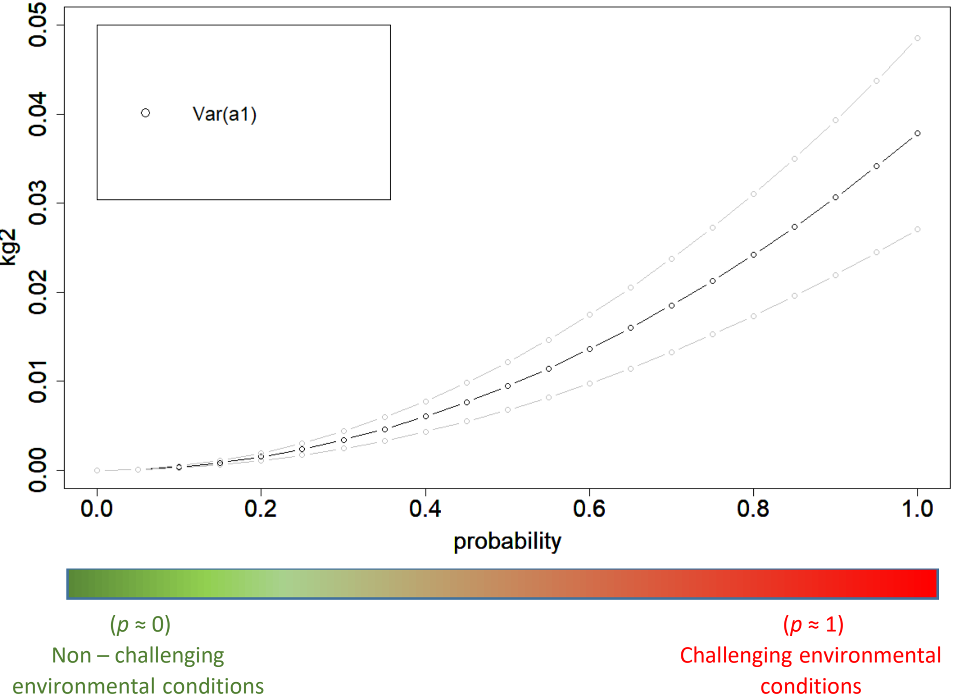
Var(a1)
kg2
0.00
0.01
0.02
0.03
0.04
0.05
0.0
0.2
0.4
0.6
0.8
1.0
probability
(p ≈ 0)
Non – challenging environmental conditions
(p ≈ 1)
Challenging environmental conditions
